# Acute Effect of Ghrelin on Ischemia/Reperfusion Injury in the Rat Spinal Cord

**DOI:** 10.3390/ijms13089864

**Published:** 2012-08-08

**Authors:** Qin Zhang, Chen Huang, Bin Meng, Tiansi Tang, Qin Shi, Huilin Yang

**Affiliations:** 1 Department of Orthopedics, Yuncheng Central Hospital, Yuncheng 044000, China; E-Mail: qinzhang_1976@163.com; 2 Department of Orthopedics, The First Affiliated Hospital of Soochow University, Soochow 215007, China; E-Mails: huangchen131@hotmail.com (C.H.); binmeng_yang@126.com (B.M.); tiansitang19762@126.com (T.T.); shishiqinqin@hotmail.com (Q.S.)

**Keywords:** ischemia/reperfusion injury, spinal cord, ghrelin

## Abstract

Ghrelin, a 28-amino acid peptide, is mainly secreted by the stomach. Ghrelin has been shown to have neuroprotective effects. However, whether ghrelin protects the spinal cord from ischemia/reperfusion (I/R) injury is unknown. To investigate this, 60 rats were randomly divided into three different groups: the sham group (*n* = 20), the vehicle group (*n* = 20), and the Ghrelin group (100 μg/kg, *n* = 20). Rats were sacrificed 12, 24, 48 and 72 h after ischemia. After the evaluation of neurologic function (48 h), the spinal cords were immediately removed for the determination of myeloperoxidase (MPO) activity (12–72 h). Apoptosis was quantitatively measured using the terminal transferase UTP nick end-labeling (TUNEL) method (24 h). The expression of bax and bcl-2 were evaluated by Western blot analysis (1 h), and *GHSR-1a* mRNA expression was detected using reverse transcriptase polymerase chain reaction (24 h). The neurological motor function was evaluated by ‘Tarlov’s score’. The neurologic outcomes in the ghrelin-group were significantly better than those in the vehicle group (*p* < 0.05). Serum tumor necrosis factor (TNF-α) levels were assessed in the peripheral venous blood. Ghrelin decreased the serum TNF-α levels and ameliorated the down regulation of spinal cord MPO activity. The expression of ghrelin receptors (GHSR-1a) in the rat spinal cord was decreased by I/R injury and increased by ghrelin. Ghrelin reduced the TUNEL-positive rate. Greater bcl-2, HSP27, HSP70, and attenuated bax expression were observed in the ghrelin-treated rats. Our results suggest that ghrelin administration may inhibit spinal I/R injury. Moreover, the improvement of neurologic function in rats was increased after the ghrelin treatment.

## 1. Introduction

Ischemic spinal cord injury is a serious complication of thoraco-abdominal aortic surgery and can cause paraplegia in approximately 40% of patients [1]. This complication has been attributed, at least in part, to temporary or permanent ischemia of the spinal cord caused by interruption of the blood supply during aortic cross-clamping [2]. Ischemia/reperfusion (I/R) injury in the central nervous system (CNS) triggers a complex series of pathophysiological events involving excitotoxicity [3], free radical production [4], inflammation [5], and apoptosis [6], all of which lead to cell death [7].

Ghrelin is a novel brain-gut peptide, a stomach hormone, secreted into the bloodstream, that initiates food intake by activating NPY/AgRP neurons in the hypothalamic acruate nucleus [8,9], and the primary endogenous ligand of the growth hormone secretagogue receptor-1a (GHSR-1a) [8–10]. Ghrelin plays an important role in both physiological and pathological processes, such as central regulation of food intake and energy homeostasis [9,11], regulation of cardiovascular actions [12], stimulation of gastric acid secretion and motility [13], inhibition of inflammation, and regulation of immune function [14,15]. Several studies have shown that ghrelin has anti-apoptotic and protective effects on various cell types subjected to I/R injury [16,17]. Recently, similar effects have been shown to be exerted in the CNS, where ghrelin inhibits apoptosis during oxygen–glucose deprivation (OGD) [18]. The effects of ghrelin on neuronal survival are not limited to neuroprotection, but also extend to cell proliferation in both embryonic and adult nervous systems [19]. However, the relationship between ghrelin and I/R injury during the acute period (1–72 h) has not been the focus of much attention. Therefore, the effect of ghrelin on the spinal cord requires further investigation. In the present study, the neuroprotective effects of ghrelin on the spinal cord are explored in an I/R rat model.

## 2. Materials and Methods

### 2.1. Materials and Methods

The study was approved by the Ethics Committee of Soochow University, China and was carried out at the Institute of Orthopedics, the First Affiliated Hospital of Soochow University. All study procedures were conducted in agreement with the guidelines for the use of experimental animals of the US National Institutes of Health. All efforts were made to minimize the quantity of animals used in the experiments and to alleviate their suffering. Also, all surgical procedures were performed in an aseptic manner and were approved by the Research Animal Resources and Care Committee of the Soochow University.

### 2.2. Animals Surgery

Sixty male Sprague–Dawley rats, weighing 300–350 g, were randomly divided into three groups: sham operation (*n* = 20), ghrelin treatment (*n* = 20) and saline treatment vehicle group (*n* = 20). Body temperature was maintained at 37 °C with an infrared heat lamp and a heating pad during the surgical procedure. Spinal cord ischemia/reperfusion was induced using the previously described method [20]. A longitudinal incision was made on the midline of the back, exposing the paravertebral muscles. Ischemia of the lumbar spinal cord was produced by occlusion of the abdominal aorta 0.5 cm below the left renal artery for 60 min, followed by 72 h of reperfusion. Following surgery, 1.0 mL saline was administered s.c. in order to replace the blood volume lost during the surgery. Sham operation rats underwent the same procedure, but no occlusion of the aorta was performed. Animals were allowed free access to water and food during recovery.

### 2.3. Drug Administration

The sham group of animals (*n* = 20) only underwent laparotomy. The vehicle group (I/R, *n* = 20) received a carrier (1 mL saline solution). The ghrelin group (*n* = 20) was injected with ghrelin (Anaspec, San Jose, CA, USA) dissolved in normal saline intraperitoneally at 100 μg/kg at the onset of ischemia.

Animals were sacrificed 12, 24, 48 and 72 hrs after ischemia (*n* = 5 per group and per time point). At each time point, animals were anesthetized with an overdose of pentobarbital and the spinal cords (T_5_–T_7_) were rapidly collected. A section of tissue was frozen in liquid nitrogen and stored at −80 °C until further use. The remaining tissue was fixed in 4% paraformaldehyde/phosphate buffered saline (PBS), followed by paraffin-embedding for sectioning.

### 2.4. Evaluation of Motor Function of Hind Limbs

An independent observer, who was blinded to the protocol and group assignments, performed the motor function assessment on rats. Grading of neurological function was performed using previously published methods from Tarlov’s score that evaluates the motor functions of the hindlimbs [21] during a 48 h observation period. The motor functions of the hind limbs were graded as: (0) complete paralysis of the hindlimbs; (1) severe incomplete paralysis of the hind limbs; (2) the hindlimbs could move but could not jump; (3) the hind limbs could jump but with obvious instability; (4) the hind limbs could jump but with slight instability; (5) the hind limbs had normal motor function.

### 2.5. Enzyme-Linked Immunosorbent Assay Measurement of Serum TNF-α

A blood sample (1 mL) was collected from the marginal ear artery of each rat prior to sacrifice. The blood was allowed to coagulate on ice and the serum fraction was separated by centrifugation at 5000 r/min for 5 minutes. Standard samples and serum samples were aliquotted into 96-well plates (TNF-α ELISA kit, eBioscience, San Diego, CA, USA) and the optical density (450 nm) was measured for each well using a microplate reader. The optical densities for each sample were compared with a standard TNF-α concentration curve created in Excel to quantify serum TNF-α.

### 2.6. Myeloperoxidase (MPO) Activity Assay with Spinal Cord Tissues

The frozen samples were weighed and a 20% homogenate was made from each sample. MPO activity was measured in each sample according to the manufacturer’s instructions (Nanjing Jiancheng Biological Institute, China) and was recorded in U/g wet tissue.

### 2.7. Terminal Deoxynucleotidyl Transferase (TdT)-Mediated dUTP Nick End Labeling (TUNEL) Staining

A TUNEL assay was conducted using a TUNEL detection kit according to the manufacturer’s instructions (Roche, Germany). Neurons with brown-stained nuclei or those containing apoptotic bodies were considered apoptotic. Independent scoring was performed by a blinded investigator and data presented as mean ± standard deviation (SD).

### 2.8. RNA Preparation and RT-PCR

The total RNA from the spinal cord was prepared with RNAiso™ Plus according to the manufacturer’s instructions (TaKaRa). Reverse transcription polymerase chain reaction (RT-PCR) was performed according to the manufacturer’s instructions (TaKaRa). The PCR reactions were carried out in a Takara gradient PCR device using a protocol consisting of 94 °C for 2 min in 1 cycle and denaturation at 94 °C for 30 s, annealing at 60 °C for 30 s, and extension at 72 °C for 60 s in each of 35 cycles for GHSR-1a and 25 cycles for GAPDH. The forward and reverse primers for rats in PCR were 5-TGGGTGTCCAGCGTCTTCTTCTTT-3 and 5-CAAACACCACCACAGCAAGCATCTG-3, respectively. The PCR products for GHSR-1a were 165 bp.

### 2.9. Western Blot Analysis

Tissue samples from SCI-injured animals were collected and homogenized on ice in 10 mM Tris–HCl buffer (pH 7.4), 10 Mm EDTA, 30% Triton-1000, 10% SDS and NaCl. Homogenates were centrifuged at 12,000 g for 30 min at 4 °C. The supernatant was collected and stored at −80 °C. Thirty micrograms of protein were size-separated by SDS-PAGE and then transferred to nitrocellulose membranes. The membranes were incubated with the primary antibodies against BAX, Bcl-2, HSP27, HSP70 and GADPH (Santa Cruz Biotechnology; Santa Cruz, CA, USA) over night at 4 °C. Then the membranes were incubated with peroxidase-conjugated bovine antirabbit IgG secondary antibody (1:2000) for 1 h at 37 °C. Immunoreactivity was detected by an enhanced chemiluminescence kit. The intensity of each band was quantitatively determined using Gel-Pro Analyzer Software (Version 4.5; Media Cybernetics, Rockville, MD, USA, 2005) and the density ratio represented the relative intensity of each band against those of controls in each experiment.

### 2.10. Statistical Analysis

All results were presented as mean ± SD. The Mann–Whitney nonparametric test was used to analyze motor function scores. One-way analysis of variance (ANOVA) was used to analyze results. Significant differences were defined as *p* < 0.05, and were determined using statistical software package SPSS 11.0 (Version 11.0; IBM: New York, NY, USA, 2011).

## 3. Results

### 3.1. Effects of Ghrelin on Motor Function of Hind Limbs

**Figure 1:**
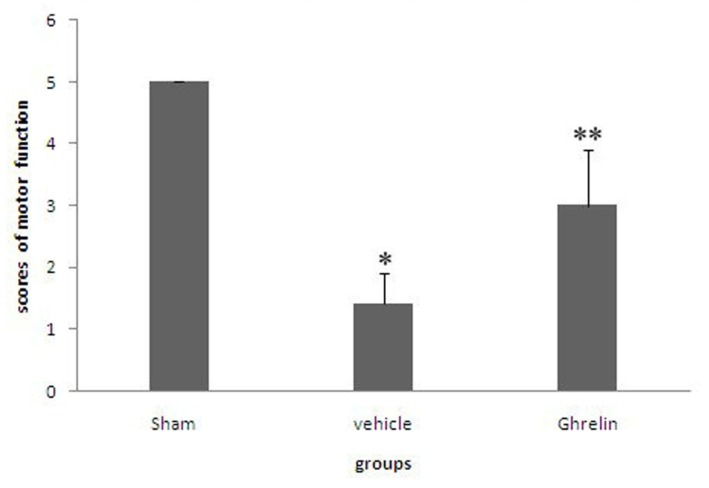
Effects of ghrelin on motor function of hind limbs. The numbers of animals in each group was 20. One rat each died in vehicle group and sham group. The degree of motor disturbance was assessed by Tarlov’s score evaluated during a 48 h observation period. Rats subjected to I/R injury showed significant effects on hindlimb movement (* *p* < 0.05). Ghrelin (100 μg/kg) reduced the degree of motor disturbances induced by I/R injury, the neurologic function was significantly better than in the vehicle group (** *p* < 0.05).

Rats that died of anesthesia or during surgery were excluded. One rat died in the vehicle group and one in the sham group. All surviving animals completed the study. The motor functions of the hind limbs of the sham-operated animals were normal. A significant distinction was observed between the two experimental groups in terms of neurological performance. Rats subjected to I/R injury showed significant effects on hindlimb movement (* *p* < 0.05). Spinal I/R resulted in significant neurological dysfunction in the saline group (Grades 0 to 2). In contrast, the rats in the ghrelin group tended to show mild neurologic deficits (Grades 3 to 4). Improvement of neurologic function was significantly better than in the vehicle group (Figure 1, ** *p* < 0.05).

### 3.2. Effects of Ghrelin Administration on TNF-α Release and MPO Activity

The serum levels of TNF-α increased from 257 to 629 pg/mL at 24 h after I/R in the vehicle-treated animals compared with the sham-operated animals (* *p* < 0.05). Administration of ghrelin significantly attenuated the serum TNF-α level. However, TNF in vehicle-treated rats was higher than in the sham-operated animals (* *p* < 0.05, Table 1). Moreover, exposure of the spinal cord to I/R resulted in a significant increase in spinal cord MPO activity (* *p* < 0.05). The treatment with ghrelin (100 μg/kg) significantly attenuated the increase in MPO activity.

### 3.3. Changes of Ghrelin Receptor in Spinal Cord

**Figure 2:**
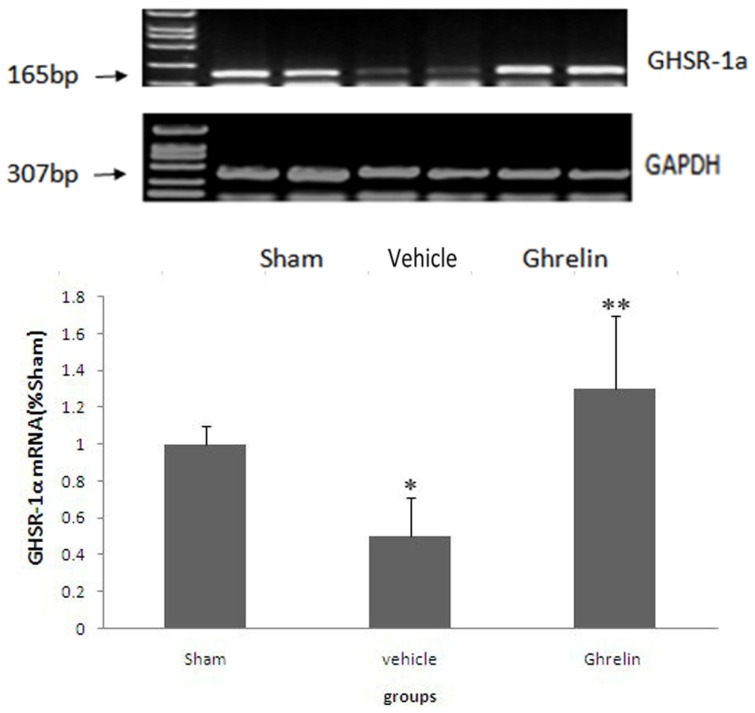
Expression of ghrelin receptor GHSR-1a in the spinal cord. Expressions of GHSR-1a were presented in the treatment groups by RT-PCR at 24 h after I/R. The GHSR-1a were 165 bp. Compared with the sham group, the I/R group showed significantly reduced GHSR-1a mRNA expression (* *p* < 0.01) that is reversed by ghrelin (** *p* < 0.05). GAPDH mRNA was also detected as an internal control (*n* = 5 per group).

Using RT-PCR, GHS-R mRNA expression was examined at 24 h after I/R in the spinal cords of rats (Figure 2). The result showed GHSR-1a mRNA expression in the spinal cord. Compared with the sham group, the I/R group showed significantly reduced GHSR-1a mRNA expression (* *p* < 0.01) that is reversed by ghrelin (** *p* < 0.05). These results suggest that the GHSR-1a level is affected by both the ischemia and ghrelin treatment.

### 3.4. Effects of Ghrelin on TUNEL Staining

**Figure 3:**
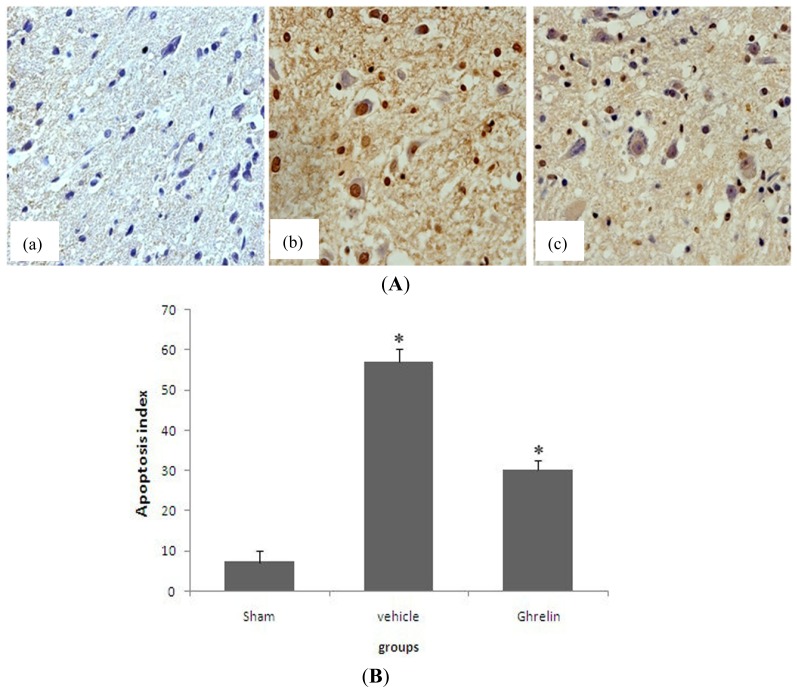
Effects of ghrelin on TUNEL staining. Ghrelin attenuated cell apoptosis in rat spinal cord ischemia/reperfusion injury by TUNEL staining at 24 h ((a) Sham group; (b) vehicle group; (c) ghrelin group). TUNEL-staining positive cells are brown in (**A**). The number of TUNEL-staining positive cells in the ghrelin group is less than in the I/R group, also, the positive cells in both the vehicle and ghrelin groups are significantly different compared with sham group (* *p* < 0.05, (**B**)) (*n* = 5 per group).

At 24 h after I/R, no TUNEL-positive (apoptotic) cells were present in the sham-operated spinal sections. A number of apoptotic cells were observed in the ischemic tissues in the saline treatment group. In the ghrelin treatment group, the number of TUNEL-positive (apoptotic) cells was significantly lower (Figure 3A). The TUNEL index showed that TUNEL-positive (apoptotic) cells were significantly lower in the ghrelin treatment group than in the vehicle treatment group (Figure 3B, * *p* < 0.05).

### 3.5. Effects of Ghrelin on Expression of HSP70, HSP27, Bax and Bcl-2

**Figure 4:**
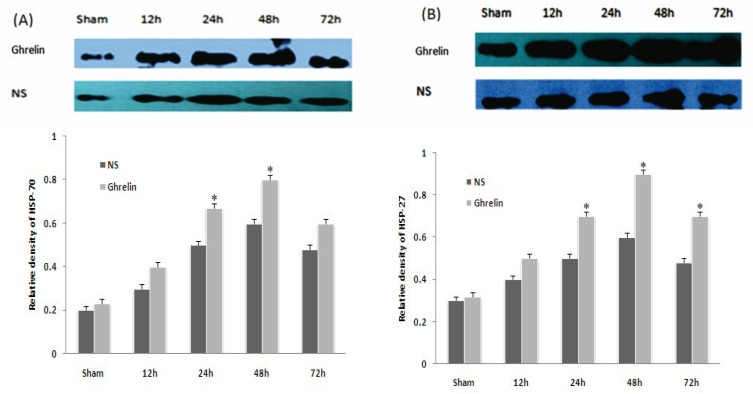
Effects of ghrelin on expression of HSP70 and HSP27. Heat shock protein 27,70 (HSP27,70) in spinal cord after 1h of ischemia followed by different times of reperfusion by Western blot. ((**A**) HSP70; (**B**) HSP27) Relative density given by the ratio of target band to GADPH band. * *p* < 0.05 *vs*. corresponding NS group. (*n* = 5 per group and per time point).

**Figure 5:**
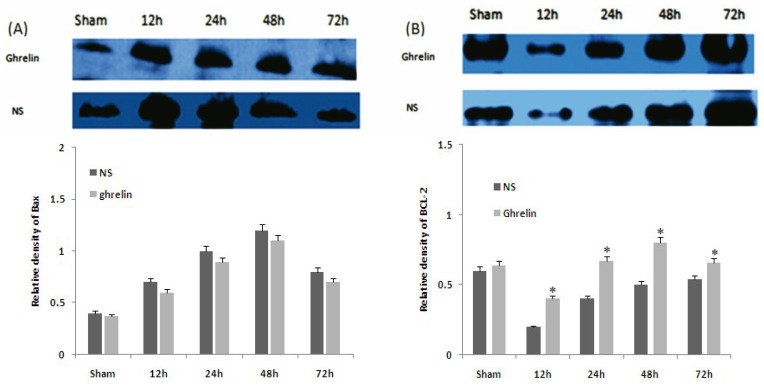
Effects of ghrelin on expression of Bax and Bcl-2. Bcl-2/Bax protein in spinal cord after 1 h of ischemia followed by different times of reperfusion by Western blot. ((**A**) Bax; (**B**) bcl-2) Relative density given as the ratio of target band to GADPH band. * *p* < 0.05 *vs*. corresponding NS group. (*n* = 5 per group and per time point).

HSP70 and HSP27 were slightly increased by I/R and were significantly increased by ghrelin administration (* *p* < 0.05, Figure 4A,B). Ghrelin administration significantly increased the level of Bcl-2 at different times after reperfusion (* *p* < 0.01), but had no significant effect on the level of Bax (Figure 5A,B). These results imply that the mitochondrial pathway is the main target of the anti-apoptotic effects of ghrelin.

## 4. Discussion

Recent studies have revealed that ghrelin receptor gene expression in the spinal cord can be detected by PCR and *in situ* hybridization [22]. In our previous work, we found the expression of GHSR mRNA in the spinal cord. Ghrelin receptor gene expression in the spinal cord decreases in the presence of I/R-induced injury, and ghrelin reverses the downregulation of GHSR-1 mRNA and protein in rat spinal cords following I/R injury.

The results of the present study revealed I/R-induced spinal cord injury, as evidenced by changes in MPO activity. Ghrelin, which demonstrated anti-inflammatory effects, protected the spinal cord against I/R-induced injury. Furthermore, the increase in serum TNF-α, which plays a pivotal role in inflammatory processes, was partially reversed by ghrelin treatment at the specific points in time. Ghrelin treatment partially protected spinal cord neurons from I/R-induced apoptosis.

MPO is an enzyme located in leukocytes. Tissue MPO levels may suggest leukocyte infiltration into spinal cord tissue after I/R injury. Neutrophils play a role in I/R-induced tissue injury by releasing various inflammatory mediators, including neutrophil elastase and oxygen-free radicals, which are capable of damaging endothelial cells [23]. Based on our findings, MPO increased in the tissue of the I/R group, but ghrelin treatment prevented this increase, thereby protecting the spinal cord. Ghrelin treatment suppressed neutrophil-dominant inflammation [24] and downregulated proinflammatory cytokines [15,25–27]. Ghrelin inhibited the I/R-induced increase in MPO activity and concomitantly abolished TNF-α response [27]. Our results suggest that neutrophil accumulation contributes to I/R-induced spinal cord injury, and the protective effect of ghrelin may be, in part, dependent on its inhibitory effect on tissue neutrophil infiltration and neutrophil-associated TNF-α response.

Apoptosis of motor neurons has been reported after transient spinal cord ischemia [28]. Mild or severe I/R injury has been shown to trigger apoptosis, which leads to cell death [29,30]. Sakurai *et al*., observed apoptotic changes in rabbit motor neurons after 15 minutes of ischemia using TUNEL staining and found that apoptosis played an important role in delayed paraplegia [31]. A recent study indicated that ghrelin protects against cell death of hippocampal neurons in pilocarpine-induced seizures in rats and hippocampal neuronal cells during oxygen-glucose deprivation [18,32]. These studies indicate that the neuroprotective effects of ghrelin are dependent on the activities of MAPK, PI3K, PKC, and PKA signaling pathways [17,18,32]. The Bcl-2 family of enzymes maintains mitochondrial stabilization to regulate apoptosis and Bcl-2(anti-apoptosis)/Bax(proapoptosis) balance, thereby determining whether cells undergo apoptosis [33]. We demonstrated that ghrelin exerted its protective action by regulating the ratio of Bcl-2 and Bax [17]. Ghrelin treatment decreased the Bcl-2 to Bax ratio. The current study shows that ghrelin significantly inhibits apoptosis of spinal cord neurons caused by I/R in rats.

HSPs prevent the aggregation and denaturation of many proteins. Endogenous protective molecules, such as HSP70, are triggered in cerebral ischemia [34] and kidney I/R injury [35]. The present study revealed that ghrelin increased the expression of HSP70 in spinal cord I/R. The role of HSPs is complex: They can be pro-inflammatory, and they may also have significant anti-inflammatory and cytoprotective actions [36,37].

In conclusion, we demonstrated GHSR-1a mRNA expression in the spinal cord and its reduction during I/R injury. The present study supports the finding that ghrelin has a partial protective effect against spinal cord I/R. The anti-apoptotic properties of the ghrelin mechanism inhibit the apoptosis molecules in the mitochondrial pathway and activate endogenous protective molecules.

## Figures and Tables

**Table 1:** Effects of ghrelin on tumor necrosis factor alpha TNF-α release and myeloperoxidase (MPO) activity. Administration of ghrelin significantly attenuated the serum TNF-α level in vehicle-treated rats at 24 h after I/R as opposed to the sham-operated animals (^*^ *p* < 0.05), however it was still higher compared to the ghrelin-operated animals (^**^ *p* < 0.05). The MPO activity was significantly increased in vehicle-treated rats, compare with sham-operated animals (^*^ *p* < 0.05). Treatment with ghrelin significantly attenuated the increase in the ghrelin-treated group compared with sham-operated animals (^**^ *p* < 0.05) (*n* = 5 per group and per time point).

Group	TNF-α (pg/mL)				

	12 h	24 h	48 h		72 h
Sham	234 ± 22.3	257 ± 12.45	185 ± 33.21		188.5 ± 10.1
Control	495 ± 5.67 ^*^	629 ± 11.98 ^*^	424 ± 50.2 ^*^		303.6 ± 18.0 ^*^
Ghrelin	378 ± 32.16 ^**^	425 ± 8.14 ^**^	343 ± 42.1 ^**^		232.2 ± 21.3 ^**^

	**MPO (U/g wet tissue)**				

	12 h	24 h	48 h	72 h	

Sham	0.31 ± 0.03	0.35 ± 0.08	0.30 ± 0.06	0.25 ± 0.01	
Control	0.92 ± 0.18 ^*^	1.53 ± 0.21 ^*^	1.09 ± 0.27 ^*^	0.75 ± 0.13 ^*^	
Ghrelin	0.44 ± 0.06 ^**^	0.88 ± 0.15 ^**^	0.78 ± 0.08 ^**^	0.32 ± 0.04 ^**^	

## List of Abbreviations

EDTA: ethylenediaminetetraacetic acid
IL-β: interleukin-1β
TNF-α: tumor necrosis factor-α
I/R: ischemia/reperfusion
MPO: Myeloperoxidase
TUNEL: terminal deoxynucleotidyl transferase-mediated UTP end labeling
HSP: heat-shock protein
PBS: phosphate buffered saline
